# What Role for Biologically Based Dose–Response Models in Estimating Low-Dose Risk?

**DOI:** 10.1289/ehp.0901249

**Published:** 2010-01-04

**Authors:** Kenny S. Crump, Chao Chen, Weihsueh A. Chiu, Thomas A. Louis, Christopher J. Portier, Ravi P. Subramaniam, Paul D. White

**Affiliations:** 1 Louisiana Tech University, Ruston, Louisiana, USA; 2 National Center for Environmental Assessment, Office of Research and Development, U.S. Environmental Protection Agency, Washington, DC, USA; 3 Johns Hopkins Bloomberg School of Public Health, Baltimore, Maryland, USA; 4 National Institute of Environmental Health Sciences, National Institutes of Health, Department of Health and Human Services, Research Triangle Park, North Carolina, USA

**Keywords:** biologically based dose response, dose-response model, low-dose risk, risk assessment, two-stage model

## Abstract

**Background:**

Biologically based dose–response (BBDR) models can incorporate data on biological processes at the cellular and molecular level to link external exposure to an adverse effect.

**Objectives:**

Our goal was to examine the utility of BBDR models in estimating low-dose risk.

**Methods:**

We reviewed the utility of BBDR models in risk assessment.

**Results:**

BBDR models have been used profitably to evaluate proposed mechanisms of toxicity and identify data gaps. However, these models have not improved the reliability of quantitative predictions of low-dose human risk. In this commentary we identify serious impediments to developing BBDR models for this purpose. BBDR models do not eliminate the need for empirical modeling of the relationship between dose and effect, but only move it from the whole organism to a lower level of biological organization. However, in doing this, BBDR models introduce significant new sources of uncertainty. Quantitative inferences are limited by inter- and intraindividual heterogeneity that cannot be eliminated with available or reasonably anticipated experimental techniques. BBDR modeling does not avoid uncertainties in the mechanisms of toxicity relevant to low-level human exposures. Although implementation of BBDR models for low-dose risk estimation have thus far been limited mainly to cancer modeled using a two-stage clonal expansion framework, these problems are expected to be present in all attempts at BBDR modeling.

**Conclusions:**

The problems discussed here appear so intractable that we conclude that BBDR models are unlikely to be fruitful in reducing uncertainty in quantitative estimates of human risk from low-level exposures in the foreseeable future. Use of *in vitro* data from recent advances in molecular toxicology in BBDR models is not likely to remove these problems and will introduce new issues regarding extrapolation of data from *in vitro* systems.

A decision regarding an acceptable level of exposure to a toxic agent can be informed by quantitative estimates of risk to humans from low exposures (e.g., exposures corresponding to increased risks of ≤ 10^−3^). Such estimates are often based on responses in animals subjected to much higher exposures. The generally accepted gold standard for making these estimates has been biologically based dose–response (BBDR) models, which incorporate information on intermediate steps in the disease process [National Research Council (NRC) 1994; U.S. Environmental Protection Agency (EPA) 2005].The U.S. EPA (2005) cancer guidelines state that “The preferred approach [to estimating low-dose risk] is to develop a toxicodynamic model of the agent’s mode of action (MOA) and use that model for extrapolation to lower doses.” However, the National Academy of Sciences (NAS) Science and Decisions Committee (NRC 2008), which was charged to develop “scientific and technical recommendations for improving risk analysis approaches used by the U.S. EPA,” did not discuss BBDR modeling. The NAS committee on Toxicity Testing in the 21 Century (NRC 2007), which reviewed toxicity testing methods and strategies and proposed a long-range vision and strategy for toxicity testing, concluded that BBDR modeling is “still in its infancy” and “the committee … does not see routine development of the models from toxicity-pathway testing data in the foreseeable future.”

BBDR models are predictive models that describe biological processes at the cellular and molecular level to link external exposure to an adverse apical response. Such models can provide estimates of the probability of an adverse response in humans, expressed as a function of quantitative biological variables involved in the adverse response. These variables (e.g., cell division rates, death rates, or production rates of hormones that govern pharmacodynamic interactions) have physiologic meaning and, at least in theory, could be measured. At least one variable must be linked to the administered dose of the toxic agent. This linkage often involves a physiologically based pharmacokinetic model that expresses exposure at a target site (e.g., tissue concentration) as a function of external exposure.

Difficulties inherent in BBDR models limit the models’ ability to provide reliable estimates of low-dose risk in humans. In view of these difficulties and of the large number of chemicals in commerce for which insufficient toxicologic information is available, large commitments of resources to complex modeling efforts with the goal of evaluating low-dose risk will not be cost effective or informative. The lessons learned from 20 years of experience with BBDR modeling should be taken into account when considering how molecular toxicology and high-throughput screening data can best be used to inform low-dose risk.

## Applications of BBDR modeling

The focus of this commentary is not on BBDR modeling per se, but more narrowly on the use of BBDR models to estimate low-dose human risk. Nevertheless, we wish to acknowledge other potentially useful applications of BBDR models. Two-stage clonal expansion models of cancer that account explicitly for mutation of normal cells to initiated cells, clonal expansion of initiated cells, and mutation of initiated cells to fully malignant cells (Moolgavkar et al. 1988a) have been used as a framework for numerous models for describing the progression of cancer (e.g., Chen and Farland 1991; Gsteiger and Morgenthaler 2008; Kopp-Schneider et al. 2005; Moolgavkar et al. 1989, 1993, 1996, 1999; Portier and Kopp-Schneider 1991; Portier et al. 1996; Tan and Chen 1998; Yang and Chen 1991). Such efforts, generally known as clonal growth models, have been useful in generating and/or evaluating hypotheses in a number of applications, thereby contributing to a better understanding of the biology. These models have also highlighted important data gaps. Examples include the carcinogenic effects of coke oven emissions (Moolgavkar et al. 1988b), diesel exhaust emissions (Chen and Oberdorster 1996), trichloroethylene (Chen 2000), refractory ceramic fibers (Moolgavkar et al. 1999), dioxin (Conolly and Andersen 1997; Luebeck et al. 1995, 2000; Moolgavkar et al. 1996; Portier and Kohn 1996; Portier et al. 1996), and formaldehyde (Conolly et al. 2003, 2004), the neurodevelopmental effects of ethanol (Gohlke et al. 2005), the developmental toxicity of 5-fluorouracil (Setzer et al. 2001), and evaluation of a hypothesized MOA for the disruption of hypothalamic–pituitary–thyroid axis homeostasis by perchlorate (McLanahan et al. 2009). We encourage continued research to evaluate potential applications of BBDR models.

## BBDR modeling has the same problems for estimating low-dose risk as empirical modeling of apical responses

Unfortunately, BBDR models have not been useful in estimating risks in the low-dose region of interest when setting exposure standards. Difficulties in using BBDR models for this purpose are conceptually the same as those faced when fitting empirical models to data on apical responses in intact animals. Moreover these difficulties are exacerbated by problems inherent in complex models.

It is well understood that exposing whole animals at low doses and measuring the apical adverse responses is of little value in informing low-dose risk, because the small change in the baseline response that one is attempting to measure is obscured by statistical uncertainty. For example, although regulatory agencies may be concerned with protecting humans against increased risks on the order of 10^−5^, the width of a 95% statistical confidence interval on increased risk in a group of 100 animals exposed to a common dose is at least 0.03, or 3,000 times greater. (For example, a 95% statistical upper bound on the probability of a response in 100 treated animals in which no response occurs is 1 – 0.05^(1/100)^ = 0.0295.) An alternative is to use an empirical dose–response model fit to apical responses obtained at high doses to estimate low-dose risk. The problems with this approach are also well understood (e.g., NRC 1983 and references therein): Different empirical dose–response models can describe the data equally well and yet predict widely different estimates of low-dose risk. It is less well understood that these exact same difficulties occur with BBDR models.

To link exposure with risk, a BBDR model must incorporate at least one biologic variable that is dose related. For example, in a model of cancer in which the mechanism is cell proliferation stemming from cytolethality, division rates of normal, but potentially tumor precursor, cells may depend on the tissue concentration of the parent chemical or a metabolite. The options for determining the low-dose response for such an intermediate variable are the same as those described above for apical responses—direct measurement or empirical modeling from high-dose data. Statistical precision is still limited by the numbers of animals. It is the between-animal variability that is important, and this cannot be improved by making many measurements (e.g., in many cells) in a few animals. Consequently, just as is true for apical responses, statistical variability will obscure efforts to measure changes in intermediate responses at doses corresponding to the small increases in risk of interest in setting of exposure standards.

The other option for estimating low-dose changes, using an empirical dose–response model fit to high-dose data on the intermediate response, has the same drawbacks as with apical responses. Although mechanistic information may indicate that a particular intermediate step is a key step toward toxicity, that information normally does not specify the form of the dose response for the intermediate variable. Consequently, dose–response modeling of that variable will be empirical, and the dose response for the apical effect will be determined by the empirical dose response assumed for the intermediate biological variable (e.g., Crump 1994a). For example, if a single intermediate variable is dose related and that variable is modeled as varying linearly, quadratically, or threshold-like at low doses, the apical response will likewise vary linearly, quadratically, or threshold-like at low doses. Thus, dose–response modeling will still be empirical, and different empirical dose–response models will fit the data on the intermediate variable equally well and yet predict widely different risks of an apical response from the same low exposures.

## BBDR modeling introduces new problems

Not only is BBDR modeling of low-dose risk subject to the same problems as empirical modeling of apical responses, but these problems are also exacerbated by uncertainties inherent in complex models and in the added data requirements and heterogeneity inherent in those data. Relevance of measurements to the mechanism in question will be uncertain. Are the responses being measured in the right cells, or are the cells at risk only those in a particular subclass or in a particular anatomical subregion? For example, data on foci or nodules have been used to estimate rates of initiation and proliferation, under the assumption that they are preneoplastic lesions. However, one cannot confidently decide which cells in foci or nodules represent initiated cells or even whether the model formulation is correct for those foci (Kopp-Schneider et al. 1998). Model conclusions can be very sensitive to these choices.

BBDR modeling of low-dose effects is greatly complicated if the toxicant affects multiple intermediate steps in the disease process. For example, in addition to affecting division rates of normal cells, the toxicant may affect division rates of initiated cells and death rates of both normal and initiated cells. But whereas the apical response can be highly sensitive to small differences in birth and death rates (e.g., Crump 1994b), measurement of these rates, if they can be measured at all, must be made in different animals, which are all different from the animals in which the apical effect was observed. In such a situation, problems of interindividual heterogeneity will magnify bounds on low-dose risk. One often-overlooked advantage of dose–response analysis directly between exposure and the frank toxic effect is that both can be measured in the same subject.

On top of the uncertainty of the relevance of the measurements to the mechanism being modeled, there are commonly important uncertainties regarding the basic mechanism itself. Current efforts by the U.S. EPA to use MOA information in risk assessment appear often to be stymied by uncertainty as to what the relevant MOA is.

## BBDR modeling uses data from the wrong species

To the foregoing problems must be added the overarching problem that most of the data for BBDR modeling come from experimental animals. Human populations differ from inbred animal strains in numerous ways, including size, longevity, genetic makeup and variability, stress factors, and exposure to infectious diseases and environmental toxicants. Consequently, the dose response for humans is possibly quite different from that for inbred animal strains. Often there is not even site concordance in tumors found in different species of rodents. Thus, even if we knew precisely the dose response in a particular animal strain, there would still be large uncertainties in extrapolating that dose response to humans. Although this uncertainty will be present in all efforts to estimate risk in humans using animal data, it argues that intensive efforts to develop data and models for BBDR modeling of specific end points observed in animals to compute low-dose risk may not be a wise use of resources.

## Illustrative examples

These problems are well illustrated by the experiences with BBDR models to date. Perhaps the most telling evidence is that, despite 20 years of effort and endorsement of such models by regulatory agencies, only a handful of BBDR models have been developed for evaluating low-dose risk, and not a single BBDR model has gained widespread acceptance for such a use. The examples discussed below are presented to illustrate the generic problems presented earlier.

Many of the BBDR modeling efforts to date do not incorporate any biological data on one or more key intermediate variables. Lacking such data, empirical dose–response models are assumed for those variables, and parameters in those models are estimated by fitting the resulting complete model to apical responses in intact animals. Such models have several potential advantages over purely empirical models, including the ability to incorporate the effect of varying dose levels in a natural way and the ability to test hypotheses about toxic mechanisms within the limits of the model. However, estimates of low-dose risk from such models are driven by the assumed empirical dose–response form(s) for the intermediate variables and consequently are subject to the same uncertainties as estimates made from empirical dose–response models fit to the apical response.

A two-stage model of lung cancer risk from exposure to refractory ceramic fibers (Moolgavkar et al. 1999) based on rat bioassay data provides a good example of such models. The final model incorporated only the mechanism of mutation from normal to initiated cells. However, no data were available on initiation, and the dose response for initiation was modeled using empirical curves whose parameters were estimated by fitting the complete model to the frank tumor data. Low-dose tumor risks predicted by a quadratic model for initiation diverged at low dose from those predicted by low-dose-linear models and were about 100-fold smaller at an exposure to 1 fiber per milliliter of air, even though the models provided similar fits to the data. This illustrates that, as pointed out earlier, in such models the estimated low-dose tumor risk is determined by the empirical form assumed for the dose response of the intermediate step(s) in the progression to toxicity.

Perhaps the most ambitious attempt to date at BBDR modeling is the model for cancer risk in the human respiratory tract from formaldehyde-induced nasal cancers observed in the F344 rat (Conolly et al. 2003, 2004). This work implemented a two-stage clonal expansion model of cancer (Moolgavkar et al. 1988a) and incorporated results of 20 years of research into formaldehyde carcinogenicity. Included in the model are data from two large rat bioassays, modeling of flux of formaldehyde into respiratory tissues in both rats and humans, data on proliferation rates of cells in the rat nasal epithelium, and data on formaldehyde- related levels of DNA–protein cross-links in rat nasal tissue. Conolly et al. (2004) claimed to provide conservative estimates of human risk, and based on the model it was concluded that cancer risks associated with inhaled formaldehyde are *de minimis* ≤ 10^−6^) at relevant human exposure levels.

However, a sensitivity analysis of this model (Conolly et al. 2009; Crump et al. 2008, 2009; U.S. EPA 2008) found that small changes to assumptions regarding the mathematical form of the dose response assumed for the division rates or death rates of initiated cells—changes too small to meaningfully degrade the correspondence of the model with the underlying data—increased estimates of cancer risk from formaldehyde by several orders of magnitude over those considered to be conservative in the original work. It is unlikely that this problem could be resolved by getting data on these rates, because the inherent variability in such data exceeds the range of the small changes made in the sensitivity analysis (Crump et al. 2008). This illustrates the inherent uncertainty in estimates of low-dose risk resulting from the uncertainty in the dose response for intermediate events.

The formaldehyde model also illustrates the formidable issues that must be faced in the difficulty of using animal data to estimate human risk. In this model, parameters estimated for rats were converted to human values by either scaling up the rat values or by simply using the value estimated from rat data. The quantitative uncertainty in such methods is difficult to estimate, but it is likely to be large, given that often there is not even site concordance between species. In the particular scaling method used in the formaldehyde model, the human risk was particularly sensitive to the background tumor rate in rats. To better estimate these background rates, Conolly et al. (2004) added historical control animals from all National Toxicology Program (NTP) bioassays to their analysis. However, the tumor rates in these added animals are statistically different from those in the historical controls drawn only from the inhalation NTP bioassays and are likely to differ from the background rate in rats in the formaldehyde bioassays because of differences in genetic makeup, handling, or living conditions. Consequently, the reduced statistical variability from adding historical controls is accompanied by potentially decreased representativeness of the control data. Indeed, different choices for control animals to include in the model can make orders of magnitude differences in the estimated low-dose risks (Crump et al. 2008; Subramaniam et al. 2007).

## General discussion

Much of the enthusiasm for use of BBDR modeling in estimating low-dose risks may result from a general aversion to the default methods contained in U.S. EPA guidelines (e.g., U.S. EPA 2005). There seems to be a sentiment that even given the uncertainty in risk estimates obtained from BBDR models, use of such models must be an improvement over default methods. We do not find this sentiment to be supported by the evidence. Although we are not claiming that the default methods consistently provide accurate estimates of low-dose risk, we see no evidence that BBDR models developed to date have reduced uncertainty in estimates of human risk or generally provided greater biological support for estimates of low-dose risk. For example, a sensitivity analysis of the formaldehyde model (Crump et al. 2008) showed that small modifications to the model that did not affect its agreement with data could produce estimates of low-dose risk ranging from negative up to values far larger than risks predicted by the U.S. EPA default method based on linear extrapolation from a point of departure (U.S. EPA 2005).

Although implementations of BBDR models for low-dose risk estimation have thus far been limited mainly to cancer modeled using a two-stage clonal expansion framework, we believe that the problems we have identified are not restricted to a particular disease or to a particular class of models, but will be present in some form in all attempts at BBDR modeling. For example, it seems unlikely that empirical modeling on some level could ever be eliminated from a BBDR model. As a result, we agree with the NAS committee (NRC 2007) that BBDR models are unlikely to be useful in low-dose risk estimation in the foreseeable future.

This conclusion seems counterintuitive: It might seem that more and better biology should result in better answers. Unfortunately, that does not seem to apply to the answers that are, at best, only inconsequentially better when applied to the problem of quantifying low-dose risk. Weinberg (1972) used the term “trans-science” to describe “questions that can be stated in scientific terms but are in principle beyond the proficiency of science to answer,” and used low-dose extrapolation to illustrate trans-science. The intervening years have only added credence to Weinberg’s judgment.

We are not recommending against research toward developing BBDR models, because such models have many potential uses. However, we recommend that before a BBDR model is used to set human exposure standards, the underlying assumptions, the choice of data, and the modeling choices be examined very carefully in view of the limitations of such models discussed here. Because critical assumptions in complex models can be hidden in the complexity, such an examination should include a thorough sensitivity analysis that evaluates the robustness of model predictions to other plausible assumptions and data interpretations. Particular care should be taken in assessing the degree of scientific support for elements in the BBDR model that critically affect low-dose risk estimates.

Future efforts to develop BBDR models for risk assessment should consider the resource and time requirements vis-à-vis the potential benefits from a BBDR model and the likelihood of success in achieving those benefits. Even if BBDR modeling to support low-dose risk estimation was superior to other approaches, the data and modeling effort required are of such a magnitude that the approach cannot be applied to more than a tiny fraction of the 82,000 chemicals now in commerce, most of which have been subjected to only very limited toxicity testing at best (NRC 2007). Widespread use of limited resources in developing BBDR models will only further exacerbate the present serious problem presented by this huge backlog of chemicals for which risk assessments are needed to support exposure standards.

In part as a possible way to address this backlog, toxicity testing is currently being revolutionized to take advantage of advances in molecular toxicology and high-throughput screening. These *in vitro* methods have the potential to allow chemicals to be tested at greatly accelerated rates and reduced costs and to reduce or even eliminate the need for testing in whole animals (Collins et al. 2008; NRC 2007; NTP 2004). Given the obvious shortcomings in the present approaches that emphasize testing at high doses in whole animals, we support efforts to move to new approaches for setting exposure standards that use *in vitro* data. Some have proposed the use of such data and computational systems biology for describing biological circuitry to develop BBDR models for estimating low-dose human risk (Conolly 2008; Kenyon et al. 2008; U.S. EPA 2006). However, we are concerned that use of *in vitro* data in complex predictive models to quantitatively inform low-dose risk will exacerbate many of the problems described herein.

## Conclusions

Although BBDR modeling held great promise as a tool that would enhance our ability to better incorporate scientific knowledge and multiple types of data into the estimation of low-dose risk, that promise has not been realized. Instead, BBDR model–based risk estimates appear to be as uncertain as those based on empirical modeling, if not more so. The problems discussed herein appear so intractable that we conclude that BBDR models are unlikely to be fruitful in reducing uncertainty in quantitative estimates of human risk from low-level exposures. Given the cost in time and effort to obtain data for these models and apply them to risk assessment, we do not see this as a sustainable approach for the future. There is, of course, still hope that this will occur, as the basic premise under which BBDR modeling was pursued for risk estimation is conceptually valid. However, it will take a technical breakthrough to meaningfully overcome the problems discussed in this paper.

Recent advances in molecular toxicology have as their strength generating understanding of the processes by which toxic agents produce their effects. Mathematical models of molecular toxicology data may make important contributions in developing this understanding. However, it is less clear how such models can be used quantitatively to inform human risk at low dose. Not only will such models be subject to the problems discussed herein, they will introduce new difficulties related to extrapolation of data from *in vitro* systems. Before committing large amounts of resources toward development of complex models from *in vitro* data for use in setting human exposure standards, we urge careful thought be given to how such models will be used and the uncertainties inherent in those models. Alternative decision-making approaches that do not require such models should also be considered.
